# Remote Monitoring for Prediction and Management of Acute Exacerbations in Chronic Obstructive Pulmonary Disease (AECOPD)

**DOI:** 10.3390/life12040499

**Published:** 2022-03-29

**Authors:** Jean-Louis Pépin, Bruno Degano, Renaud Tamisier, Damien Viglino

**Affiliations:** 1 HP2 Laboratory, Grenoble Alpes University, INSERM U1300, 38000 Grenoble, France; bdegano@chu-grenoble.fr (B.D.); rtamisier@chu-grenoble.fr (R.T.); dviglino@chu-grenoble.fr (D.V.); 2 EFCR Laboratory, Thorax and Vessels Division, University Hospital of Grenoble Alpes, 38043 Grenoble, France; 3 Emergency Department, University Hospital of Grenoble Alpes, 38043 Grenoble, France

**Keywords:** acute exacerbations of COPD, remote monitoring, prediction, telemedicine, integrated management

## Abstract

The progression of chronic obstructive pulmonary disease (COPD) is characterized by episodes of acute exacerbation (AECOPD) of symptoms, decline in respiratory function, and reduction in quality-of-life increasing morbi-mortality and often requiring hospitalization. Exacerbations can be triggered by environmental exposures, changes in lifestyle, and/or physiological and psychological factors to greater or lesser extents depending on the individual’s COPD phenotype. The prediction and early detection of an exacerbation might allow patients and physicians to better manage the acute phase. We summarize the recent scientific data on remote telemonitoring (TM) for the prediction and management of acute exacerbations in COPD patients. We discuss the components of remote monitoring platforms, including the integration of environmental monitoring data; patient reported outcomes collected via interactive Smartphone apps, with data from wearable devices that monitor physical activity, heart rate, etc.; and data from medical devices such as connected non-invasive ventilators. We consider how telemonitoring and the deluge of data it potentially generates could be combined with electronic health records to provide personalized care and multi-disease management for COPD patients.

## 1. Introduction

Chronic obstructive pulmonary disease (COPD) leading to increased morbidity and early mortality is a major public health problem with more than 3 million deaths globally each year. The World Health Organization estimates that COPD will become the third leading cause of death worldwide by around 2030 [1]. Individual COPD trajectories are punctuated by acute exacerbations of COPD (AECOPDs) characterized by sudden worsening in the patient’s symptoms, acceleration of decline in respiratory function, deterioration in quality of life, and an increase in healthcare consumption [2,3]. These acute exacerbations represent major events in the progression of the disease and are the dominant cause of mortality.

COPD exacerbations are triggered by environmental factors including respiratory infections and air pollution [4]. Also, weather conditions including unusually low temperatures have been shown to be significantly associated with the occurrence of AECOPD. The impact of such external factors has recently been evidenced by a 50% reduction in hospitalization for COPD exacerbations during the COVID-19 pandemic compared to the pre-pandemic period. This essentially corresponded to reduced exposure to respiratory viral infections that are common triggers of exacerbations [5]. Beyond the COVID-19 pandemic, strategies to reduce infections such as wearing masks and physical distancing might have a considerable impact in preventing AECOPDs.

The frequency of exacerbations varies greatly among COPD phenotypes and patient profiles. Anthropometrics and COPD-related risk factors depicting the frequent exacerbator phenotype (≥2 exacerbations per year) have been consistently reported in previous studies [4,6,7,8,9]. COPD exacerbations are more common in females, patients with cardiometabolic or psychiatric comorbidities—in particular depression—and at the most severe spectrum of the disease. A history of prior exacerbations has been demonstrated to have by far the strongest association with the risk of future exacerbations [7,8].

Overall, owing to the major impact of AECOPD on individual patient’s trajectories and the burden put on the health system and society, COPD exacerbations constitute the major focus of COPD routine care. In addition to strategies to reduce the risk of a COPD exacerbation, there is an urgent need to challenge the usual therapeutic inertia characterized by under-recognition of the early stages of AECOPD and inappropriate delays before initiating or escalating adequate therapies [10,11]. The management pathway should be reshaped with the overarching goals of the timely identification of relevant multifaceted alerts, improvement in risk prediction by including environmental factors, continuous assessment of disease activity, and knowledge of treatable traits in a given COPD patient [10]. This will allow proactive, early, and integrated interventions, potentially reducing costs and the morbi-mortality of patients with AECOPD.

The integrated management of severe COPD patients and the early detection of acute exacerbations requires considerable human resources and caregivers face difficulties in personalizing the management of follow-up pathways. A major step forward is the recent availability of mobile health tools [12] and remote monitoring platforms [13,14], which can provide daily information on external factors that trigger AECOPDs and enable the longitudinal collection of data characterizing a patient’s physiological and clinical status. Such digital medicine solutions—by upgrading the detection of acute events—might represent an attractive option for improving the early identification of AECOPDs.

**Figure 1:**
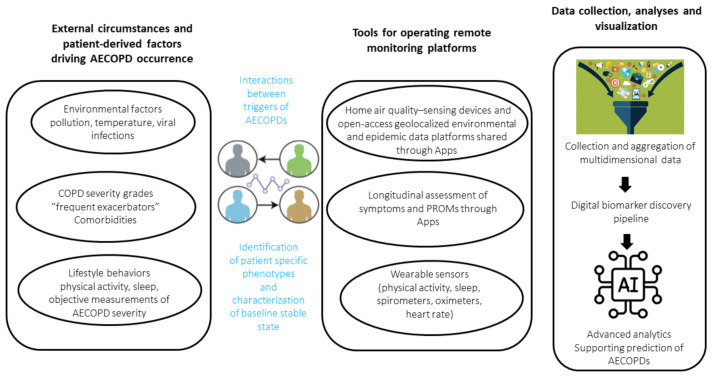
Factors driving the occurrence of AECOPD, tools for monitoring these factors, and data collection, analyses, and visualization.

In the present review, we aim to summarize the recent scientific data on remote telemonitoring (TM) of acute exacerbations in COPD patients, and whether these solutions meet expectations. We also look at how to design the different components of remote monitoring platforms and their performances, challenges, and pitfalls. Lastly, we explore how TM and the deluge of data it potentially generates could be combined with electronic health records to provide personalized care and multi-disease management for COPD patients (Figure 1).

## 2. Designing Telemonitoring Platforms for Early Identification of AECOPDs

The generic term “exacerbation” does not reflect the diversity and the complexity of AECOPDs [9]. AECOPDs have multiple causes, often in combination, and occur in distinct phenotypes. The COPD “frequent exacerbator” phenotype is consistently defined by at least two treated exacerbations per year and is associated with poor long-term outcomes and an accelerated decline in lung function. An early identification strategy based on the detection of variations in the burden of external factors and adapted to at-risk clinical profiles might enhance AECOPD detection.

Lessons learnt from the literature provide guidance regarding the items to be collected for accurately predicting COPD exacerbations via mobile health solutions, connected devices, or remote monitoring platforms. The overarching goal is to assess the combination of contextual external factors and patient-derived information.

### 2.1. Assessment of External Factors

Environmental data on the patient’s everyday exposures—such as pollution, temperature, circulating viruses—can be collected from air quality-sensing devices at home and/or from various open-access environmental data platforms [15]. Such relevant information can be shared through apps with COPD patients engaged in their own self-management owing to frequent exacerbations of their disease [16]. A patient’s knowledge of at-risk environmental situations will enable them to make informed choices and modify their own risk of exacerbation by limiting social interactions, avoiding polluted areas, and asking family members to prudently wear masks in case of infection symptoms [17]. There are many smartphone applications now providing information regarding actual indices of air quality with appropriate spatial and temporal granularities. The main barriers to take-up of such prevention measures are age, education, and socioeconomic status.

### 2.2. Specific Questionnaires to Identify Patients at High Risk of AECOPDs and the Longitudinal Collection of Patients’ Symptoms 

The first step is to define the population of “frequent exacerbators” who need to be given priority when deploying costly, educational, time consuming, and complex remote monitoring solutions. There are large variations in the risk of exacerbation across COPD patients and in routine clinical practice a history of two or more exacerbations is classically the most robust predictor of future exacerbations. Frequent exacerbators also exhibit more airflow reduction, symptoms, and variations in health-related quality of life [7]. However, even in this subgroup a considerable disparity in risk persists and additional tools for stratification of vulnerability are required. The Acute COPD Exacerbation Prediction Tool (ACCEPT) has recently been validated using data from three randomized trials to predict the rate and severity of COPD exacerbations [8]. ACCEPT provides—at an individual level—a risk profile that might help clinicians to tailor strategies for including patients in programs for the early prediction of exacerbations. The ACCEPT profile cannot be manually calculated as the total score requires complex computational methods [8]. However, the score calculation is readily accessible using a web or smartphone application (https://CRAN.R-project.org/package=accept, accessed on 28 March 2022). This could facilitate the dissemination of ACCEPT in routine care and will allow it to be included in remote monitoring platforms. In the long run the goal is to automatically establish patient-specific critical baseline profiles to prioritize the prediction of exacerbation in clinically meaningful populations [18].

Once a patient is assigned to a high-risk group for severe acute exacerbation of COPD, a procedure for the collection of patient-reported outcomes can be installed on a smartphone, downloaded, and amassed on a regular basis [19] along with longitudinal data on the evolution of symptoms. A recent multicenter study has prospectively recorded daily symptoms over a 6-month period in a cohort of 116 COPD patients through the smartphone app, Prevexair^©^ [16]. Acceptance of the daily data collection was high (i.e., more than two-thirds of the participants) and the study demonstrated good performances for the early detection of COPD exacerbations [16]. For confirmation of an acute exacerbation and improvement in specificity and sensitivity, many remote monitoring solutions are including objective measurements of lifestyle changes [20] (physical activity [15] and/or sleep) reflecting deterioration in clinical status or physiological parameters that directly reveal the severity of AECOPD (cough sound [19], breathing frequency, SpO_2_, heart rate, etc.).

### 2.3. Wearable Sensors Automatically Capturing Lifestyle Data (Physical Activity, Heart Rate, and Sleep Patterns)

Changes in everyday activities are good indicators of deterioration in respiratory function. There is a clear relationship between exacerbation symptoms and a reduction in physical activity. A mean decrease of 700 steps per day was associated with an increase in the EXACT score indicating the start of an exacerbation [21]. This underscores the importance of corroborating symptoms with their objective impact on everyday activities. Breathlessness, cough, and sputum also affect sleep structure and quality. It has been reported that higher scores in the Pittsburgh Sleep Quality Index (PSQI) are associated with daytime symptoms that enhance the probability of AECOPDs [22].

### 2.4. Remote Patient Monitoring Technologies for the Detection of COPD Exacerbations

There are many handheld and hands-free monitors available, including spirometers, pulse oximeters, and electronic inhalers [23]. These devices allow monitoring of lung function, deterioration in gas exchanges, and adherence to medication(s). A large spectrum of wristband or watch-like monitors include different combinations of physiological signals, namely breathing frequency, heart rate, blood pressure, physical activity, and sleep. Costs are highly variable and the validation of clinical reliability is poorly established. There is still debate regarding the interest of once daily versus multiple point measurements of physiological parameters. It has been demonstrated that overnight pulse oximetry increases sensitivity and allows earlier detection of COPD exacerbations compared with once-daily monitoring [20].

Specific treatment options for extremely severe COPD are available, including long-term oxygen therapy and chronic noninvasive ventilation. Every day these home-treatment devices can provide relevant information for detecting AECOPDs. It has been demonstrated that respiratory rate, monitored daily at home in patients receiving domiciliary oxygen therapy, increases significantly a few days before they require hospitalization because of AECOPDs [24]. Parameters recorded by software of non-invasive ventilators can also be used to predict COPD exacerbations [25]. An increase in respiratory rates and the percentage of respiratory cycles triggered by the patients nearly systematically preceded exacerbation in patients with COPD treated by home NIV [25]. These data have been replicated in another independent study [26] and home ventilation machines can now wirelessly transmit data for remote monitoring in routine practice [27,28]. Data from home spirometry and oximeters can complement ventilator data in this high-risk population [28].

Another important aspect is to monitor the time course of recovery following an acute AECOPD event so as to make a timely decision for when to discharge from hospital and avoid early readmission [29]. The same tools can be used to characterize transition from stable to prodromes of exacerbations and different recovery trajectories requiring or not additional interventions.

## 3. Data Analysis, Artificial Intelligence, and Visualization Tools

The prediction of AECOPDs using telemedicine is at the confluence of remote sensing, patients’ utilization of personal technologies, and data processing and analysis supported by various artificial intelligence (AI) methods facilitating medical decision making [12]. The initiation of such complex integrated systems cannot be driven only by technological innovation, but their design should be shared with expert clinicians and users, i.e., patients. More and more, patient groups that by definition are “experts by experience” are implicated in the proposal and validation of remote monitoring platforms [18]. A partnership with patients is crucial when developing a remote monitoring platform. Such a co-design should include structured patient interviews, health professional focus groups, patient co-creation activities, and health professional prioritization discussions. After several iterations, the proposed solution is shared with all stakeholders to test a prototype to be validated. Further efforts should focus on the continued development and testing of the integrated care platforms with a prospective collection of outcomes. Patients’ experiences and long-term adherence could also be improved through the visualization of relevant online information and patients’ engagement tools.

The challenge is to implement and sustain a system architecture for the prediction of AECOPDs including three components, detailed in the following sections.

### 3.1. Data Collection and Aggregation

The target is to collect patient-reported outcomes, physiological data, and external environmental data sets via apps and connected devices [18]. Data can be collected automatically via Bluetooth, directed to web platforms or customized apps [15], or assembled using application programming interfaces (APIs) to avoid physicians or other caregivers having to log into multiple systems for every sensor or app included in a patient’s monitoring [12]. In a perfect world, the remote monitoring platform allowing prediction of AECOPDs would merit being linked to the electronic medical records (EHR) of the patients’ healthcare institutions [12].

### 3.2. Visualization Tools to Engage Patients and Physicians 

The reports of data should be appealing with the presentation of key information by visualization on dashboards that can be actualized in real time. Dashboards need to be shared with patients through a smartphone [30] and provided to clinicians as part of the clinical workflow through a window embedded within the EHR.

### 3.3. Data Processing and Analysis 

To be fully exploited, such a massive data stream with a combination of external and bio-clinical data sets requires reference data-analysis methodologies and the validation of automated algorithms. Different machine learning-based classification and deep neural networks are now at the core of features identification and analyses. The competencies and experience of clinicians should also be used to complete supervised learning and the identification of features of interest [15]. This collaborative work with expert caregivers must also define relevant thresholds for AECOPDs or regulation based on clinical experience [18]. Some teams [18] have worked to define a baseline stable state for given patients which permits differentiation between intra- and inter-person variability and the improved detection of AECOPDs [18]. In real-life observational data, the problem of missing values is crucial, as patients may or not follow the requirements for regular data acquisition via apps and connected devices. To address this issue, some authors have developed prediction via optional features when only incomplete daily data are automatically uploaded [15]. Finally, already impressive results have been published with a combination of patient-reported outcomes and reliable bio-physiological inputs, allowing performances above 85% for sensitivity and specificity in predicting AECOPDs to be achieved [18].

### 3.4. Allocation of Resources

The developed decision support systems provide “red flags” suggesting the onset of exacerbation [14] and the direct assessment by clinicians would generate an overly challenging workload. A pre-selection of significant alerts must be ensured by automated procedures and allocated to case managers or home care providers for triage. This raises the pivotal question of cost-effectiveness and appropriate reimbursement models.

## 4. Effectiveness of Remote Monitoring Interventions for Detection and Management of AECOPDs: Data from Randomized Controlled Trials and Observational Studies

A Cochrane review [31] has summarized the impact of remote monitoring technology for people with COPD. Most of the included studies required participants to transfer measurements using a remote sensor allowing an asynchronous review by a caregiver. Overall, the quality of existing studies was poor with levels of evidence ranging from moderate to very low [31]. The combination of remote monitoring with usual care (eight studies, 1033 participants) had little to no effect on the number of people experiencing exacerbations or on hospitalization rates [31]. Remote monitoring demonstrated a possible positive impact on readmissions after hospitalizations. There was no evidence of harm with these telehealth interventions. Another systematic review reported more positive perceptions [32]. Remote monitoring of COPD patients was considered effective in reducing emergency department presentation. Indeed, remote patient monitoring was more effective in COPD than in other chronic disease conditions [32]. Of the 13 RCTs included in the systematic review, 30% reported a reduction in hospital use and all cohort studies (n = 9) were positively in favor of remote monitoring [32].

## 5. Challenges, Pitfalls, and Perspectives of Remote Monitoring for the Prediction of AECOPDs

The next priorities in the field are to address barriers to bringing remote monitoring platforms into clinical validation in real-world medicine settings and implementation in healthcare services [33,34].

### 5.1. Prioritization of Items and Tools to Be Included in AECOPDs Remote Monitoring Platforms

The best combination of patient reported outcomes (PROMS) and data generated by apps and sensors to be finally included in remote monitoring platforms is difficult to choose. Monitoring symptoms and PROMs are probably as accurate as physiological variables for the prediction of AECOPDs and might represent a simpler approach. On the other hand, measurements of objective physiological parameters allow the strict verification of true exacerbations, thus increasing the specificity and validity of alerts, and with good patient compliance such data provide individual benefit to the use of the system [30]. It is still unclear which COPD severity subgroups would benefit most from telehealth interventions, and the complexity of system architectures and management follow-up pathways should probably be tailored according to phenotype and baseline risk stratification.

In the medical community, there are misperceptions concerning the different wearable devices dedicated to wellness, healthcare, or research applications [33]. However, business models and partnerships are distinct in these different contexts, and beyond technological choice, it should be anticipated how the remote monitoring platform will become financially and logistically sustainable [33].

### 5.2. Validation of Remote Monitoring Platforms for AECOPDs Prediction

The diversity of physiological and lifestyle sensors on the market is rapidly evolving, requiring the continuous renewal of remote monitoring platform tools and requiring incessant adaptations in artificial intelligence techniques to maintain accurate decision support tools. These unceasing technological and analytic innovations complexify the design and the running of appropriate validation studies. Randomized controlled trials are probably no longer the best way to evaluate these digital health solutions. As in other chronic diseases such as sleep apnea, huge, real-life, longitudinal cohorts generating large observational datasets are well adapted to provide guidance regarding the impact of digital health and the best configuration of management pathways [35,36,37]. The observational nature of the data and specificities of datasets generated by wearable sensors with potential population selection bias and a large amount of missing data create new methodological issues [38]. These problems can be solved, and new and relevant pipelines for digital marker discovery have been implemented [39] along with innovative data generation [40].

### 5.3. Long-Term Adherence with Remote Monitoring Platforms

There is little literature specifically addressing patient adherence in the daily use of remote monitoring tools [16]. Over time, there is clearly a progressive disinterest if the platform does not sufficiently communicate with patients and does not use interactive engagement tools. Patients who continue to smoke and who have the worst lifestyle habits and/or depressive profiles are the most likely to have intermittent use of their remote monitoring tools [16]. Technology literacy is the ability to effectively use technology and represents a key issue for the dissemination of remote telemonitoring [41]. Patients lacking the abilities and confidence to use technology are likely to be left behind, leading to health disparities and an inability to be included in integrated telemedicine follow-up.

### 5.4. Factors Influencing the Accessibility, Uptake, and Effectivesness of Remote Monitoring

Finally, data privacy, ethical, and disparity considerations along with patient-related factors such as socio-economic status, age, health insurance, dependency, geographic location, education level, language, and race are true concerns, but beyond the scope of this review [33,42,43].

## 6. Conclusions and Perspectives

AECOPD features are challenging to predict via remote monitoring platforms because they present very differently among diverse COPD patient phenotypes and are related to many external factors. However, a combination of patient reported outcomes and data generated by wearables, supported by artificial intelligence prediction models, permits the development of more robust prediction algorithms. Policymakers should now include these remote monitoring platforms in reimbursed care pathways and define the respective roles of caregivers in managing alerts and integrated care. These digital solutions also increase the patients’ engagement in their own care, autonomy, and quality of life. They could also help to identify factors associated with the onset of AECOPD and thus guide therapies to prevent future exacerbations.

## Data Availability

Not applicable.
